# Genotoxicity of nano/microparticles in *in vitro *micronuclei, *in vivo *comet and mutation assay systems

**DOI:** 10.1186/1743-8977-6-23

**Published:** 2009-09-03

**Authors:** Yukari Totsuka, Takashi Higuchi, Toshio Imai, Akiyoshi Nishikawa, Takehiko Nohmi, Tatsuya Kato, Shuich Masuda, Naohide Kinae, Kyoko Hiyoshi, Sayaka Ogo, Masanobu Kawanishi, Takashi Yagi, Takamichi Ichinose, Nobutaka Fukumori, Masatoshi Watanabe, Takashi Sugimura, Keiji Wakabayashi

**Affiliations:** 1 Cancer Prevention Basic Research Project, National Cancer Center Research Institute, 1-1 Tsukiji 5-chome, Chuo-ku, Tokyo 104-0045, Japan; 2 Central Animal Laboratory, National Cancer Center Research Institute, 1-1 Tsukiji 5-chome, Chuo-ku, Tokyo 104-0045, Japan; 3 Division of Pathology, National Institute of Health Sciences, 1-18-1 Kamiyoga, Setagaya-ku, Tokyo 158-8501, Japan; 4 Division of Genetics and Mutagenesis, National Institute of Health Sciences, 1-18-1 Kamiyoga, Setagaya-ku, Tokyo 158-8501, Japan; 5 Department of Food and Nutritional Sciences, Graduate School of Nutritional and Environmental Sciences, University of Shizuoka, 52-1, Yada, Shizuoka, 422-8526, Japan; 6 Fundamental Nursing, School of Nursing, University of Shizuoka, 52-1, Yada, Shizuoka, 422-8526, Japan; 7 Environmental Genetics Laboratory, Frontier Science Innovation Center, Osaka Prefecture University, 1-2 Gakuen-cho Naka-ku, Sakai-city, Osaka, 599-8570, Japan; 8 Department of Health Sciences, Oita University of Nursing and Health Sciences, 2944-9 Megusuno, Oita-city, Oita, Japan; 9 Department of Environmental Health and Toxicology, Tokyo Metropolitan Institute of Public Health, 24-1, Hyakunin-cho 3-Chome, Shinjuku-ku, Tokyo, 169-0073, Japan; 10 Division of Materials Science and Engineering, Yokohama National University, Graduate School of Engineering, 79-5, Tokiwadai, Hodogaya-ku, Yokohama, 240-8501, Japan

## Abstract

**Background:**

Recently, manufactured nano/microparticles such as fullerenes (C_60_), carbon black (CB) and ceramic fiber are being widely used because of their desirable properties in industrial, medical and cosmetic fields. However, there are few data on these particles in mammalian mutagenesis and carcinogenesis. To examine genotoxic effects by C_60_, CB and kaolin, an *in vitro *micronuclei (MN) test was conducted with human lung cancer cell line, A549 cells. In addition, DNA damage and mutations were analyzed by *in vivo *assay systems using male C57BL/6J or *gpt *delta transgenic mice which were intratracheally instilled with single or multiple doses of 0.2 mg per animal of particles.

**Results:**

In *in vitro *genotoxic analysis, increased MN frequencies were observed in A549 cells treated with C_60_, CB and kaolin in a dose-dependent manner. These three nano/microparticles also induced DNA damage in the lungs of C57BL/6J mice measured by comet assay. Moreover, single or multiple instillations of C_60 _and kaolin, increased either or both of *gpt *and Spi^- ^mutant frequencies in the lungs of *gpt *delta transgenic mice. Mutation spectra analysis showed transversions were predominant, and more than 60% of the base substitutions occurred at G:C base pairs in the *gpt *genes. The G:C to C:G transversion was commonly increased by these particle instillations.

**Conclusion:**

Manufactured nano/microparticles, CB, C_60 _and kaolin, were shown to be genotoxic in *in vitro *and *in vivo *assay systems.

## Background

Nano/microparticles are widely used because of their desirable properties in industrial, medical and cosmetic fields [1-6]. Accordingly, these particles can be released into the human environment and then can be inhaled. Most exposure to airborne nano/micromaterials occurs in the work place. Nano/microparticles can be classified into three groups: natural, anthropogenic and man-made (or artificial). The natural kind, for example, is produced during forest fires or volcanic eruptions. Anthropogenic particles are quite often a by-product of industrial activities such as welding or polishing. Diesel exhaust products, PM10 and PM2.5, well known as combustion nanoparticles, also belong to this group. The man-made group includes engineered nanomaterials [5].

Among these nano/mocroparticles, diesel exhaust particles have been well documented, in their general toxicity, mutagenicity and carcinogenicity [7-10]. In addition, asbestos, a naturally occurring nano-sized silicate mineral fiber, has been considered to be a human carcinogen [11-13]. Animal experiments and epidemiological studies have already demonstrated that pulmonary fibrosis, bronchogenic carcinomas and malignant mesotheliomas are closely associated with asbestos exposure. Another mineral fiber, titanium dioxide (TiO_2_) has also been subjected to extensive research, and TiO_2 _has already been shown to be carcinogenic [14]. Moreover, man-made vitreous fibres, including glass fibres, refractory ceramic fibres, and rock wool, have been sorted as carcinogens [15]. Kaolin/kaolinite is a clay mineral with the chemical composition Al_2_Si_2_O_5_(OH)_4_, and is used in ceramics, medicines, food additives, toothpaste and cosmetics. The largest use of kaolin is in the production of paper [3]. In 1993, W. B. Bunn 3rd *et al*. reported that increased incidences of lung tumors and mesotheliomas were observed in long-term inhalation studies of rats and hamsters treated with micro-sized refractory ceramic fibres containing kaolin as the main component [16]. However, other genotoxic and carcinogenic potentials of kaolin have not been studied *in vitro *and *in vivo*. In addition, the mechanism of cancer development by kaolin is still unclear.

On the other hand, carbon black (CB), fullerenes (C_60_) and carbon nanotubes (CNTs) are developed as engineered nanoproducts [1,2,6,17]. Despite their highly desirable structures, their toxicity and carcinogenicity are concerns because these engineered nanoproducts are considered to be very stable and could lead to continuous inflammation when deposited in tissues. CNTs especially have received much attention from the aspect of toxicity due to their asbestos-like rod-shaped particles, and iron content [17-19]. Recently Takagi *et al*. demonstrated that multi-wall carbon nanotubes induced mesothelioma in *p53*+/- mice by a single i.p. injection [20]. In contrast, C_60 _is a spherical molecule consisting entirely of carbon atoms, and various derivatives have been reported [6,21,22]. C_60 _has widely different properties, such as scavenging of reactive oxygen species, direct interaction with biomolecules and radical formation; however, clear genotoxic and carcinogenic effects have not yet been demonstrated.

The present study aims to examine the genotoxicity/clastogenicity of widely distributed nano/microparticles such as C_60_, CB and kaolin by an *in vitro *micronucleus test. Moreover, we analyzed the genotoxic effects of these particles by an *in vivo *comet assay and mutation assay system using *gpt *delta transgenic mice. In this mouse model, point mutations and deletions are separately analyzable by *gpt *and Spi^- ^selections, respectively [23,24]. The mutation assay using the *gpt *delta mouse was validated and so far is widely used in the field of environmental mutagenicity.

## Results

### Size distribution and agglomeration state in suspensions of nano/microparticles

**Figure 1:**
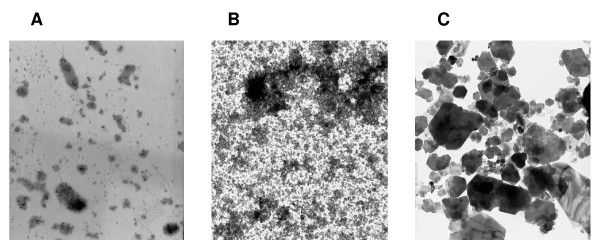
**Representative TEM images of the presently used nano/microparticles within the suspensions**. C_60 _(PanelA), CB (Panel B) and kaolin (Panel C) were suspended in saline containing 0.05% Tween 80 at a concentration of 2 mg/mL with a 10 min sonication. All images are shown at the original magnification of × 10,000.

Figure 1 shows representative transmission electron microscope (TEM) images for the state of test materials dispersed in saline containing 0.05% Tween 80. These were commonly observed to be a mixture of well dispersed fine particles and agglomerates. C_60 _was frequently agglomerated, but fine particles were also observed either individually or within pear-shaped agglomerates. In contrast, CB was relatively well dispersed, and agglomerates were occasionally present. In the case of kaolin, low-density tabular structures with rectangular or hexagonal shape were characteristically observed. The size distribution of materials used in the present study was analyzed by dynamic light scattering (DLS). C_60 _demonstrated a wide distribution with ranges of 10.5 to 12913.9 nm, and most abundant sizes were two peaks at 234.1 ± 48.9 and 856.5 ± 119.2 nm, respectively. CB particles formed a normal distribution with ranges of 13.6 to 337.4 nm and major peak average was at around 232.0 nm. In the case of kaolin, a major peak average was 357.6 ± 199.4 nm belonging to a range of 5.1 to 4846.9 nm. Although the primary particle size of kaolin was 4.8 μm, it is likely that sonication might lead to size reduction.

### *In vitro* micronucleus test

**Figure 2:**
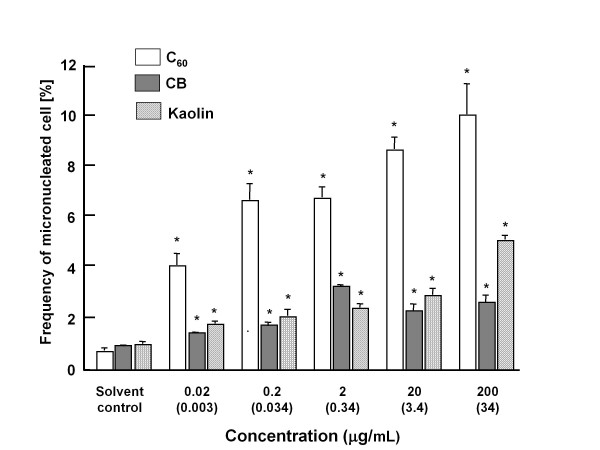
**Frequency of micronucleated A549 cells incubated with C_60 _CB or kaolin**. The values represent the mean of three experiments ± SD. An asterisk (*) represents that each frequency is significantly different (*p *< 0.01) from that of control cell in a Student's t-test. Concentrations in μg/cm^2 ^are given in parenthesis.

To examine the genotoxicity of particles, we analyzed the micronucleus inducing activity of C_60_, CB and kaolin using human lung cancer cell line, A549. A six-hour treatment of 200 μg/mL CB and kaolin caused growth inhibition of 60% in A549 cells; however, C_60 _did not inhibit growth of cells at any concentrations (between 0.02 - 200 μg/mL, data not shown). As shown in Figure 2, C_60 _and kaolin particles increased the number of micronucleated cells in a dose-dependent manner. On the other hand, CB increased the number of micronucleated cells up to 2 μg/mL, and thereafter seemed to plateau. The background frequency of micronucleated cells was 0.7% to 1.0%, and the frequency rose to 10% and 5% at 200 μg/mL of C_60 _and kaolin, respectively, and 3.3% at 2 μg/mL of CB treatment. The increase of the frequency from that of the control cells was statistically significant in all particle-treated cells. C_60 _demonstrated the most strong genotoxic/clastogenic potencies among these three particles.

### *In vivo* genotoxicity analyzed by alkaline comet assay

**Figure 3:**
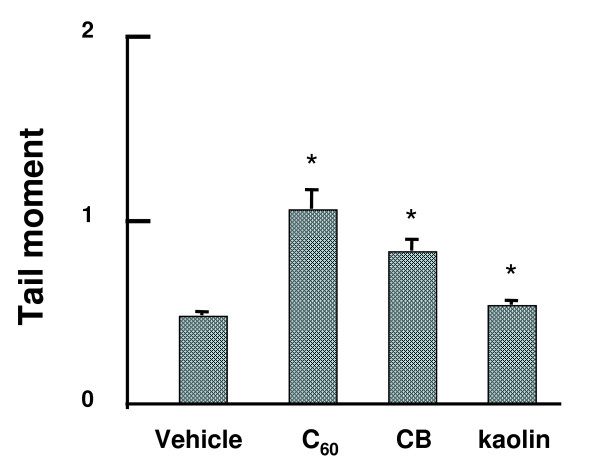
**DNA damage in lungs of C57BL/6J mice intratracheally instilled with particles**. DNA damage was measured by comet assay. Male mice were treated at a dose of 0.2 mg per animal of particles, and mice were sacrificed 3 hr after particle administrations. The values represent the mean of five animals ± SE. An asterisk (*) denotes *p *< 0.01 in a Dunnett's test after one-way ANOVA of Tail Moment of particle-treated vs. corresponding vehicle-control mice.

DNA damage induced by particles was evaluated using comet assay under alkaline conditions. Figure 3 shows the mean values of DNA tail moment in the lungs with or without single-particle treatment at 0.2 mg/body for 3 hr. In the case of particle exposure, DNA damage was significantly increased as compared with the vehicle control up to 2 - 3 fold, and its intensity was C_60 _> CB > kaolin. On the other hand, we examined the genotoxicity of nano/microparticles at a dose of 0.05 mg/animal. DNA damage observed in the lung of mice was almost the same as those of the vehicle control (data not shown). Moreover, we examined the effects of different exposure times for 3 and 24 hr. While DNA damages induced by CB or kaolin were not changed either for 3 or 24 hr, DNA damage caused by C_60 _was decreased for 24 hr compared with 3 hr (data not shown). It seems that DNA damage repair enzymes might affect the result of comet assay.

### General observations of *gpt* delta transgenic mice administrated with particles

Body weights of *gpt *delta mice receiving a single dose of vehicle control reached 31.1 ± 1.8 g at 12 weeks after instillation. Values for *gpt *delta mice which received a single dose of particles at 0.2 mg/body were 30.0 ± 2.4 g for C_60_, 32.6 ± 1.1 g for CB and 30.8 ± 2.3 g for kaolin, respectively, at 12 weeks after instillation. The average consumption of diet per day per mouse was 3.6 g, with no effects from particle instillation. No body weight and diet consumption changes were also observed with multiple doses of particles. All mice used for the single dose study survived to the end of the study, although, in the case of multiple doses, one fullerene- and one kaolin-administrated mouse died within two weeks after the last instillation, probably due to respiratory disturbances.

### *gpt* Mutations in the lungs of *gpt* transgenic mice with particle treatment

To determine the mutagenic effects of particles in the lungs, *gpt *delta transgenic mice were exposed to C_60_, CB and kaolin at doses of 0.2 mg/body by single intratracheal instillation, and mutations were analyzed. Figure 3 shows the mutant frequencies (MFs) of the lungs. The background MF of lungs was 10.30 ± 0.53 × 10^-6^. MFs in the lungs induced by C_60 _and kaolin were significantly increased by 2-fold compared with vehicle-instilled animals. CB showed increasing tendency for MF in the lungs, but not statistically significant.

**Figure 4:**
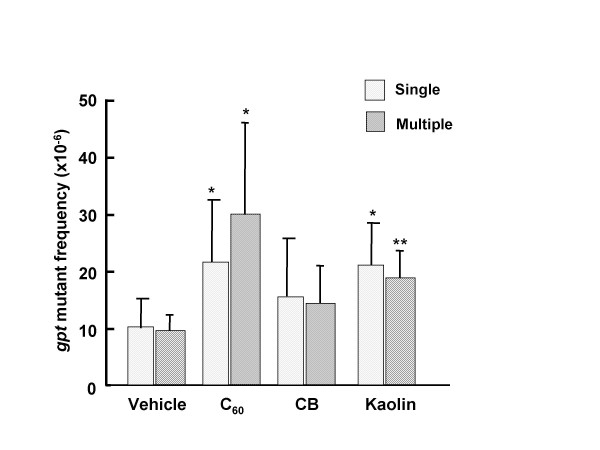
***gpt *MFs in the lungs of mice singly and multiply intratracheally instilled with particles**. Male mice were treated with single (0.2 mg per animal) or multiple (0.2 mg per animal × 4) doses of particles, and mice were sacrificed 12 (single) and 8 (multiple) weeks after particle administrations. Mean values ± SD are shown. An asterisk (*, **) denotes *p *< 0.05 (*) and *p *< 0.01 (**) in a Student's *t*-test of MF of particle-treated vs. the corresponding vehicle-control mice.

Next, we examined the mutagenic effects of consecutive exposure of particles. The *gpt *MFs in the lungs obtained from mice multiply exposed (4 times) to 0.2 mg/body each of C_60_, CB or kaolin are shown in Figure 4. In cases of C_60 _and kaolin, MFs of the lungs were significantly higher as compared to those of control animals, and their values were 2 - 3 fold increased. In the case of CB exposure, MFs were slightly increased but not statistically significant.

To analyze the mutational characteristics induced by particles, we examined PCR and DNA sequencing analysis of 6-thioguanine (6-TG)-resistant mutants. More than 40 independent 6-TG resistant mutants derived from multiple particle instillation (0.2 mg × 4), and 25 mutants from vehicle instilled animals were identified. Classes of mutations found in the *gpt *gene are listed in Table 1. Base substitutions predominated with both particle-induced and spontaneous cases. No A:T to T:A and G:C to C:G transversions were detected in vehicle control groups, indicating that these types of mutations are rare events in the spontaneous mutations. Interestingly, G:C to C:G transversion commonly increased in all three particle treatments compared to the vehicle control. G:C to A:T transition also significantly increased in CB and kaolin instillation but not in C_60_. In addition, the numbers of A:T to T:A transversion were slightly increased in the treatment with C_60 _and CB. Other types of mutations, including deletions and insertions, were also observed in both particle-treated and vehicle control groups, but these were of minor significance.

**Table 1:** Classification of *gpt *mutations from the lungs of control and particle multiply (0.2 mg × 4) treated mice^a)^

	Control		C_60_		CB		Kaolin	
	
Type of mutation in gpt	No.	%	No.	%	No.	%	No.	%
Base substitutions								
Transitions	10	40	35	41	18	45	37	50
A:T->G:C	2	8	11	13	2	5	5	7
G:C->A:T	8	32	24	28	16	40	32	43
Transversions	10	40	35	40	17	43	30	41
A:T->T:A	0	0	2	2	1	3	0	0
A:T->C:G	2	8	3	3	4	10	5	7
G:C->T:A	8	32	25	29	8	20	17	23
G:C->C:G	0	0	5	6	4	10	8	11
Deletions	4	16	12	14	4	10	6	8
Insertions	1	4	3	4	0	0	1	1
Others	0	0	1^b)^	1	1^C)^	3	0	0
Total	25	100	86	100	40	101	74	100

^a)^Independent mutations were isolated no more than once from any individual mouse.

^b)^Multiple mutation (Four base substitutions)

^C)^Tandem mutation (GG->TT)

**Figure 5:**
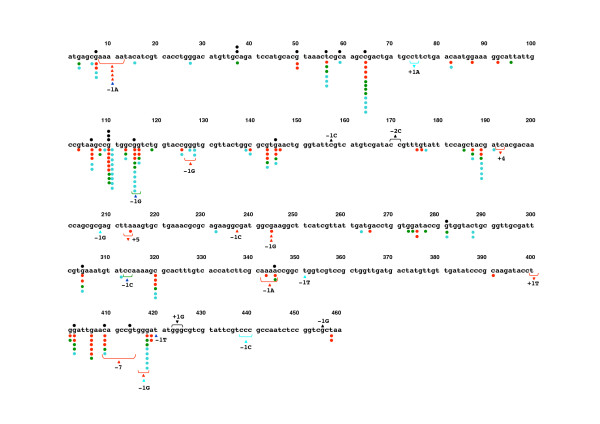
**Spontaneous and particle-induced mutations in the coding region of the *gpt *gene**. Mutations obtained from the control mice are shown above the wild type sequence, and mutations obtained from the particle-treated mutant clone are shown below the wild type sequence. The types of particles are indicated by color coding: red for C_60_, blue for CB and sky blue for kaolin. Mutation types, base substitution, and deletion and insertion are indicated by circle, triangle, and inverted triangle, respectively.

The distribution of spontaneous and particle-induced mutations in the *gpt *gene is shown in Figure 5. Base substitutions were spread throughout the coding region with a preference for some sites. However, clear mutational hotspots for each particle could not be seen except deletion mutations occurring at a run of 5 adenines (positions 8 to 12) and at position 244 for C_60 _treatment. The distribution of base substitutions along the *gpt *gene did not vary with the particle types. Twelve out of 200 particle-induced mutations occurred at position 64, eighteen at position 110, ten at position 115. All of the base substitutions occurring at positions 110 and 115 were G to A transitions, and at position 64 were C to T transitions, which were common among spontaneous mutants. In contrast, four to eight mutations occurred at positions 116, 143, 189, 320, 406 and 418 were only seen in the particle-treated mice, therefore it is suggested that these mutations can be considered as particle-induced mutations. Among these, five out of six mutations at position 406 were found in C_60 _instillation, and all mutation patterns were G to T transversions. Four out of 7 and five out of 8 at positions 189 and 418 were detected in kaolin instillation, and the majorities of the mutations were G to A and C to A, respectively. Moreover, these hotspots induced by particles occurred at G or C residues in the *gpt *gene without association for specific sequences.

### Spi^- ^MFs in the lungs of *gpt* transgenic mice with particle treatment

**Figure 6:**
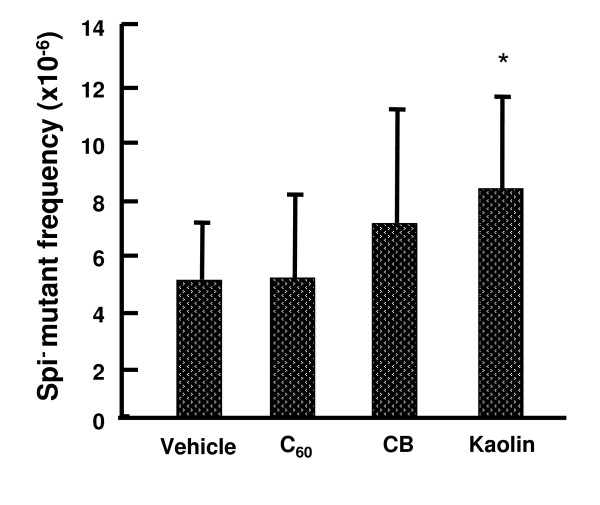
**MFs of deletions in the lungs of *gpt *delta mice exposed to multiple doses of particles**. An asterisk (*) denotes *p *< 0.05 in a Student's *t*-test of MFs of particle-treated vs. the corresponding vehicle-control mice.

We also measured the Spi^- ^MFs in the lungs of *gpt *delta mice instilled with multiple doses (0.2 mg × 4) of particles (Figure 6). Spi^- ^MFs of the vehicle control was 4.85 ± 2.04 × 10^-6^, in contrast, particle-administrated groups were 4.91 ± 3.03 × 10^-6 ^for C_60_, 6.87 ± 4.06 × 10^-6 ^for CB and 8.12 ± 3.32 × 10^-6 ^for kaolin. As shown in Figure 6, Spi^- ^MFs in the lungs of the CB- and kaolin-treated, but not C_60_-treated groups were increased, and in particular, the values of the kaolin-treated groups were significantly elevated up to 2-fold.

### *gpt* Mutations in the kidneys of *gpt* transgenic mice with particle treatment

To determine the tissue distribution and specificity of particles with intratracheal instillation, *gpt *MFs of the kidney were analyzed. *gpt *MFs of the vehicle control versus particle-multiple administrated groups (0.2 mg × 4) were 1.33 ± 0.51 × 10^-5 ^versus 1.67 ± 0.66 × 10^-5 ^for C_60_, 1.03 ± 0.39 × 10^-5 ^for CB and 1.32 ± 0.32 × 10^-5 ^for kaolin. From these observations, it is suggested that these particles did not induce mutation in the kidneys under these conditions.

### Histopathological evaluation

**Figure 7:**
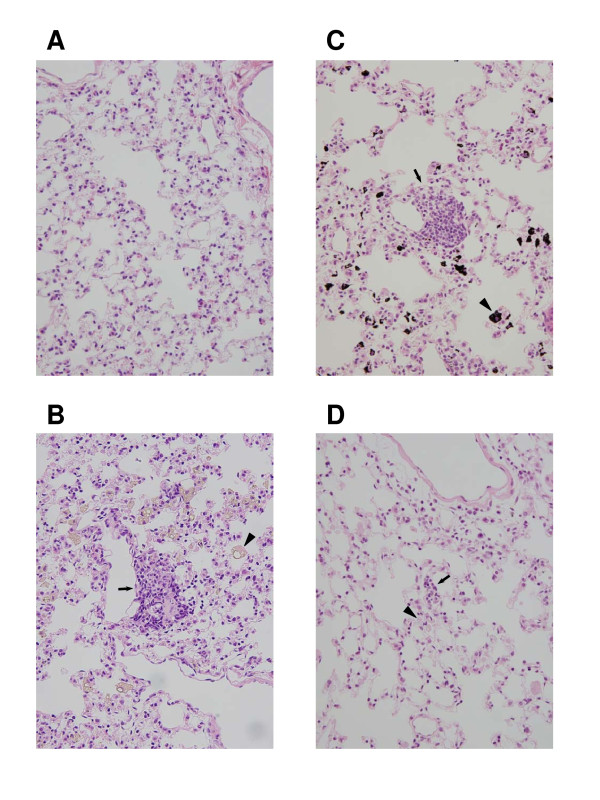
**Microscopic findings in lungs of *gpt *delta mice intratracheally instilled with particles**. Normal appearance of pulmonary parenchyma in a vehicle-control (Panel A). Pulmonary parenchyma obtained from *gpt *delta mice intratracheally instilled with four consecutive doses of 0.2 mg/mice of C_60 _(Panel B), CB (Panel C) and kaolin (Panel D). Test substance-phagocytized macrophages (arrowheads) can be observed, and granulomaous (arrows) formations are also found in lungs of particle-instilled mice. A-D; Original magnification × 40.

Histopathological analyses of lung tissues of *gpt *delta mice consecutively instilled particles, C_60_, CB and kaolin, at 0.2 mg/body per week for 4 weeks each are shown in Figure 7. Test substances-phagocytized alveolar macrophages were diffusely found in the lungs, but not in the vehicle group. Focal granulomatous formation accompanied with or without the test substance-phagocytized macrophages were also frequently observed in the lungs of particle-multiply-instilled mice. Similar findings, but a slight degree of particle accumulation and granuloma formation, were also observed in lungs of mice with particle single-instillations (data not shown). The degree of granuloma formation in the lungs of multiple C_60_- or CB-exposed mice appeared more severe than those in multiple kaolin-exposed mice. No abnormalities were observed in the kidneys obtained from mice multiply instilled with particles (data not shown).

## Discussion and conclusion

This study demonstrated the genotoxicity of nano/microparticles widely used for industrial, cosmetic and medical fields. In *in vitro *genotoxic analysis, increased MN frequencies were observed in A549 cells treated with C_60_, CB and kaolin in a dose-dependent manner. On the other hand, these three particles also induced DNA damage in the lungs of C57BL/6J mice measured by comet assay. Furthermore, we found that C_60 _and kaolin demonstrated mutagenicity either or both of *gpt *and Spi^- ^mutations in the *gpt *delta transgenic mice systems. The *gpt *gene MFs were significantly increased in the lungs of *gpt *delta mice with C_60 _and kaolin, but not CB administrations. A dose-dependent MF increase was observed in the lungs of C_60_, but not kaolin treated groups. The reason is still unclear, but suggesting that the single dose of kaolin already represented the maximum response. On the other hand, kaolin demonstrated significantly increased Spi^- ^MFs; however, C_60 _showed similar values compared with the vehicle control of the lungs. Spi^- ^selection detects deletions in size more than 1 bp and 10 kb [24]; therefore, additional DNA damages involved in deletion mutations might be induced by kaolin. It, it is also suggested that C_60 _does not prefer to induce such kinds of DNA damages under these conditions. In contrast to the present study, Xu *et al*. have reported that C_60 _dramatically increases large deletion mutations in *gpt *delta transgenic mouse primary embryo fibroblast cells [25]. The observed difference of mutational signatures of C_60 _between a cell line and lung tissue might be related to differences between *in vitro *and *in vivo *assay systems in DNA damage formations, DNA repair or translesion DNA synthesis.

To further elucidate the mechanisms behind the increase in mutant frequency observed in this study, we analyzed mutation spectra using a PCR-direct sequencing method. Most mutations induced by three particles in the present study, occurred at G:C base pairs (52/76, 68%). Among these, 13 G:C base pairs were located in the G or C runs. The most prominent hot spots were at base pairs 143, 189, 320, 406 and 418, and there were no significant differences in the distributions of mutation hot spots in the three particles. This may reflect the distribution of DNA damage sites caused by particles. The most prominent mutation type induced by particles was G:C to C:G transversion. Since these mutations were commonly increased regardless of the constituents of particles (i.e. C_60 _and CB were graphite and kaolin was aluminum silicate), it is suggested that mechanisms leading to the induction of such kinds of mutations might be same. In general, the G:C to C:G transversion is thought to be a rare event in both spontaneous and chemically-induced mutations. However, various oxidative stresses caused by sunlight, UV radiation, hydrogen peroxide and peroxy radicals frequently induce G:C to C:G transversion in *in vitro *assay systems [26-29]. Reactive oxygen species (ROS) and DNA damage, including 8-oxo-7,8-dihydro-2'-deoxyguanosine (8-oxo-dG), were reported to be increased by nanoparticles, including asbestos, treatment [4,21,30-34]. The mechanism of the generation of ROS by nanoparticles is still unclear; however, these nanoparticles would be able to trigger ROS production by iron-catalysed Fenton reactions, or would be accumulated in the cells by phagocytosis, then enhance the production of ROS from macrophages and leucocytes [35,36]. In the present study, test substance-phagocytized macrophages and granulomas were frequently observed in the lungs, and the degree of the granulomas formation was partly associated with the mutagenic effect on *gpt *gene by particles. In the case of C_60_, generation of ROS along with lipid peroxidation via electron transfer between C_60 _and other molecules has been reported [21]. The most typical lesion of oxidative damage is 8-oxo-dG which can pair with dA and leads G to T transversions [37,38] but it is not responsible for G to C transversion since dG is not incorporated opposite 8-oxodG [37,39]. Moreover, a variety of oxidative lesion products of guanine other than 8-oxodG, including imidazolone (Iz), oxazolone (Oz), spiroiminodihydantoin (Sp) and guanidinohydantoin (Gh), have been reported [39-45]. Recently, three such molecules, Oz, Sp and Gh are thought to be the key molecules causing G to C transversion using the translesion synthesis systems [43-46]. Moreover, these molecules have also been detected in bacterial cells and rat liver [47,48]. Therefore, it is suggested that G:C to C:G transversions induced by particles such as C_60_, CB and kaolin could involve Oz, Sp and Gh formations.

In the present study, G:C to A:T transition and A:T to T:A transversion were also increased in the particle treatment. G to A transition has commonly been observed in spontaneous and chemically-induced mutants and deamination of 5-methylcytosine or alkylation of guanine might be involved in these mutations. In contrast to G to A transition, A:T to T:A transversion is known as a rare mutation. It has been reported that the most common mutations induced by N-ethyl-N-nitrosourea in the mouse are A:T to T:A transversions [49]. However, at present, the mechanisms underlying generation of A to T transversion by particles are still unclear.

As mentioned above, we found that all three particles, C_60_, CB and kaolin increased significant DNA damage in the lungs compared to the vehicle control using the comet assay. Comet assay under alkaline conditions is used to detect both strand breaks and DNA altering lesions such as an AP site [50]. Moreover, in the present study, treatments with C_60_, CB and kaolin significantly increased the frequency of micronucleated A549 cells in a dose-dependent manner. However, these genotoxic/clastogenic potencies did not necessarily correspond to the mutagenicity observed in *gpt *transgenic mice.

In conclusion, we demonstrated that manufactured nano/microparticles such as C_60_, CB and kaolin were shown to be genotoxic in both *in vitro *and *in vivo *assay systems. Moreover, it was not necessarily the case that genotoxic potency was related to particle size (C_60 _and CB are nano-sized, but kaolin is micro-sized particles used in the present study.). From the prominent mutation spectra, it is suggested that oxidative DNA damage might be commonly involved in their mutagenicity. The dose of particles used in the present study seems to be extremely high compared with human exposure in the work place. However, it is likely that these materials would be deposited for a long time in tissues, same as those of asbestos fiber. Therefore, further studies of the mechanisms of genotoxicity and application routes other than trachea are needed. Moreover, exposure levels of these genotoxic particles in the working environment should be determined.

## Materials and methods

### Materials and chemicals

CB nanoparticles with a primary particle size of 14 nm (Printex 90) were obtained from Degussa, Dusseldorf, Germany. The surface area was 300 m^2^/g (disclosed by Degussa). The CB was autoclaved at 250°C for 2 h before use. High purity (99.9%) C_60 _was purchased from Sigma-Aldrich. (St. Louis, MO, USA). The declared primary particle size of C_60 _was 0.7 nm. Kaolin, white crystal, with a primary particle size of 4.8 μm was obtained from Engelhard Corp., Iselin, NJ. C_60_, CB and kaolin particles were suspended in saline (Otsuka Pharmaceutical Co. Ltd., Tokyo, Japan) containing 0.05% of Tween 80 (Nacalai Tesque, Kyoto, Japan) by sonication for 15 - 20 min, at a concentration of 2 mg/mL. The size distributions of the presently used nano/microparticles in the suspensions were measured by dynamic light scattering (DLS) using FPAR-1000 (Otsuka electronics Co., Ltd., Osaka), and the agglomeration state was assessed by transmission electron microscope (TEM) (H-7000, Hitach, Ltd., Tokyo, Japan). The size distributions were determined with the algorithm CONTIN. For the TEM assessment, an aliquot of 5 μL was put on the nickel glid coated by hydrophilized formbar and assessed with an accelerating voltage of 75 kV.

Type I agarose, low melting point agarose, dimethylsulfoxide and Triton X-100 were bought from Sigma-Aldrich. Ethidium bromide was obtained from Merck (Darmstadt, Germany). Other chemicals were purchased from Wako Pure Chemical Industries (Osaka, Japan).

### Micronucleus test

Human lung carcinoma A549 cells obtained from the RIKEN Cell Bank (Wako, Japan) were cultured in Eagle's minimum essential medium (Nissui Pharmaceutical Co. Ltd., Tokyo, Japan) supplemented with 10% fetal bovine serum (JRH Biosciences, Lenexa, KS, USA) in a 5% CO_2 _atmosphere at 37°C. The cells (7 × 10^5 ^cells/dish) were seeded in plastic cell culture dishes (φ60 mm) one day before treatment. Particles were suspended in physiological saline containing 0.05% (v/v) Tween-80 with sonication (for 5-10 min at room temperature). One volume of the suspension was mixed with 9 volumes of the culture medium with serum (altogether 3.3 mL/dish), and then cells were treated at indicated concentrations for 6 hr. Since a long exposure (48 hr) increased the frequency of micronucleated cells in the solvent control (data not shown), we chose a 6 hr treatment. After treatment, cells were further cultured for 42 hr. Then, cells were trypsinized and counted, and centrifuged. Growth inhibition was calculated by following the formula:



Cells were resuspended in 0.075 M KCl, and incubated for 5 min. Cells were then fixed 4 times in methanol:glacial acetic acid (3:1), and washed with methanol containing 1% acetic acid. Finally, cells were resuspended in methanol containing 1% acetic acid. The cell solution was dropped onto slides and the nucleus was stained by mounting with 40 μg/mL acridine orange (Nacalai Tesque) solution and immediately observed by fluorescence microscopy using blue excitation. The number of cells with micronuclei was recorded based on observation of 1,000 interphase cells. The data of EMS and mitomycine C (MMC) for positive system controls in CHL cells under the same experimental conditions were as follows; Percentage of micronucleated cells were 9.8 ± 0.68 for EMS (1 mg/mL) and 10.3 ± 1.1 for MMC (100 n/mL), respectively.

### Animals

Male C57BL/6J mice (9 weeks old) were purchased from Charles River Japan, Inc. (Atsugi, Japan) and *gpt *delta mice (9 weeks old) were obtained from Japan SLC (Shizuoka, Japan), respectively. The *gpt *delta mice carry approximately 80 copies of *lambda *EG10 DNA on each chromosome 17 on a C57BL/6J background [23]. Animals were provided with food (CE-2 pellet diet, CLEA Japan, Inc., Tokyo, Japan) and tap water *ad libitum *and quarantined for one week. Mice were maintained under controlled conditions: 12-h light/dark cycle, 22 ± 2°C room temperature, and 55 ± 10% relative humidity. The experiments were conducted according to the "Guidelines for Animal Experiments in the National Cancer Center" of the Committee for Ethics of Animal Experimentation of the National Cancer Center.

### Treatment of wild type and *gpt* delta transgenic mice with particles

All particles were well sonicated and suspended in saline containing 0.05% of Tween 80. For comet assay, 5 male C57BL/6J mice were intratracheally instilled with particles using a polyethylene tube under anesthesia with 4% halothane (Takeda Chemical, Osaka, Japan). Single doses of 0.05 or 0.2 mg per animal were employed. The control mice (n = 5) were instilled intratracheally with 0.1 mL of the solvent alone. The mice were sacrificed 3 hr after these particle administrations, and lungs were removed then used for comet assay immediately. In addition, different exposure time (24 hr) was also examined. For histological and mutation analysis, each group of 10 male *gpt *delta mice was intratracheally instilled with particles at a single dose of 0.2 mg per animal, and multiple doses of 0.2 mg per animal per week for 4 consecutive instillations, as described for comet assay. The intratracheal instillation dose of particles between 0.05 and 1 mg/mouse has been commonly used for the pulmonary inflammation and genotoxicity test [51,52]. The control mice (n = 10) were instilled intratracheally with the solvent alone. The mice were sacrificed at 22 weeks old being 12 (for single instillation) or 8 (for multiple instillations) weeks after particle administrations, respectively. Tissues, including lungs and kidneys, were removed. Lungs and kidneys obtained from 4 mice were used for histological evaluation and examined under a light microscope for any abnormalities. For histopathological evaluation, organs were fixed in 10% neutral buffered formalin, embedded in paraffin blocks and routinely processed to H&E stained sections. The remaining 6 mice were used for mutation analysis and the tissues were stored at -80°C until the DNA was isolated.

### Alkaline comet assay

The alkaline comet assay was performed according to the method of Sasaki et al. [53] or Toyoizumi et al. [54] with some modification. The lungs were taken from treated mice and weighed, and lung tissue was minced and suspended with chilled homogenizing buffer, then homogenized gently using a Dounce-type homogenizer in ice.

Lung cell suspension was mixed with the same volume of 1.4% low melting point agarose in PBS. The mixture was layered on the slide coated with 0.7% agarose layer, and then covered with 0.7% low melting point agarose. After slide preparation, slides were immersed in lysing solution and refrigerated at 4°C for 1 h. Each slide was then placed in alkaline electrophoresis buffer for 10 min to allow for DNA unwinding. Electrophoresis was performed at 25 V, 300 mA for 15 min at 0°C. The slides were neutralized with Tris buffer for 5 min twice, and dehydrated with 70% ethanol to fix. The cells were stained with ethidium bromide solution. Comet images were analyzed using a fluorescence microscope (magnification 200×) equipped with a CCD camera. Fifty cells were examined per mouse. The tail moment of DNA was measured using Comet Analyzer Youworks Bio Imaging Software.

### *gpt* and Spi^- ^mutation assays

High-molecular-weight genomic DNA was extracted from the lungs and kidneys using a RecoverEase DNA Isolation Kit (Stratagene, La Jolla, CA) according to the instruction manual provided by the supplier. *Lambda *EG10 phages were rescued using Transpack Packaging Extract (Stratagene).

The *gpt *mutagenesis assay was performed according to previously described methods [55]. Briefly, *E. coli *YG6020 was infected with the phage and spread on M9 salt plates containing Cm and 6-TG, then incubated for 72 hr at 37°C. This enabled selection of colonies harboring a plasmid carrying the gene for chloramphenicol acetyltransferase, as well as a mutated *gpt*. Isolate exhibiting the 6-TG-resistant phenotype was cultured overnight at 37°C in LB broth containing 25 mg/mL Cm, then harvested by centrifugation (7,000 rpm, 10 min), and stored at -80°C.

The mutation spectrum of 6-TG cording sequence were performed by PCR and direct sequencing. Briefly, a 739 bp DNA fragment containing *gpt *was amplified by PCR as described previously [30,53]. Sequencing analysis was done at Takara Bio Inc. (Mie, Japan).

The Spi^- ^assay was performed as described previously [53]. The lysates of Spi^- ^mutants were obtained by infection of *E. coli *LE392 with the recovered Spi^- ^mutants. *gpt *and Spi^- ^MFs were determined in each mouse and the means ± standard deviations were calculated.

### Statistical analysis

The data from micronucleus test and *gpt *and Spi^- ^mutation assay are expressed as mean ± standard deviations. The data obtained from comet assay are expressed as mean ± standard errors. The data were statistically compared with the corresponding solvent control using the Student's t-test for micronucleus and *gpt *and Spi^- ^mutation assay. To test for significant differences of tail moment in the comet assay between a group treated with materials and an untreated group, Dunnett's test after one-way ANOVA was used to evaluate the differences; *p *values lower than 0.05 were considered to indicate statistical significance.

## Abbreviations

CB: carbon black; C_60_: fullerenes; MN: micronuclei; CNTs: carbon nanotubes; TEM: transmission electron microscope; DLS: dynamic light scattering; MFs: mutant frequencies; 6-TG: 6-thioguanine; 8-oxo-dG: 8-oxo-7,8-dihydro-2'-deoxyguanosin; Iz: imidazolone; Oz: oxazolone; Sp: spiroiminodihydantoin; Gh: guanidinohydantoin; ROS: reactive oxygen species.

## Competing interests

The authors declare that they have no competing interests.

## Authors' contributions

YT carried out the preparation and performance of *gpt *delta transgenic mouse experiments and drafted the manuscript. SO and MK performed *in vitro *MN tests. TK and SM performed the comet assay. TI, KH and TH performed the animal exposure and *gpt *and Spi^- ^mutation analysis. Pulmonary and renal histopathological evaluations were done by TI and AN. Analysis of size distribution and agglomeration state of particles were done by MW and NF. TN, NK, TY, TS and KW conceived and supervised the study. All authors read and approved the final manuscript.
